# Pharmaceutical Industry Evaluation of the Effectiveness and Efficiency of the ZaZiBoNa Collaborative Medicines Registration Initiative: The Way Forward

**DOI:** 10.3389/fmed.2022.898725

**Published:** 2022-04-25

**Authors:** Tariro Sithole, Gugu Mahlangu, Stuart Walker, Sam Salek

**Affiliations:** ^1^School of Life and Medical Sciences, University of Hertfordshire, Hatfield, United Kingdom; ^2^Medicines Control Authority of Zimbabwe, Harare, Zimbabwe; ^3^Centre for Innovation in Regulatory Science (CIRS), London, United Kingdom; ^4^Institute for Medicines Development, Cardiff, United Kingdom

**Keywords:** ZaZiBoNa, regulatory harmonisation, work sharing, effectiveness, efficiency, common technical document (CTD)

## Abstract

**Introduction:**

The common technical document (CTD) format harmonised the requirements for the registration of medicines, which had traditionally differed from country to country, making it possible for countries to collaborate and conduct joint reviews of applications. One such collaborative medicines registration initiative is the Southern African Development Community ZaZiBoNa, established in 2013. A recent study was carried out with the nine active member regulatory authorities of the ZaZiBoNa to determine their views on its operational effectiveness and efficiency. Having obtained the authorities’ views, the aim of this study was to evaluate the effectiveness and efficiency of the current operating model of the ZaZiBoNa initiative including the challenges it faces as well as identifying opportunities for improvement from the applicants’ perspective.

**Methods:**

Applicants who had submitted registration/marketing authorisation applications for assessment under the ZaZiBoNa initiative during 2017–2021 were recruited into the study. Data was collected in 2021 using the Process, Effectiveness and Efficiency rating questionnaire (PEER-IND) developed by the authors. The questionnaire was completed by a representative responsible for ZaZiBoNa submissions in each company.

**Results:**

The pharmaceutical industry was of the view that the ZaZiBoNa initiative has achieved shorter timelines for approval of medicines, resulting in increased availability of quality-assured medicines for patients in the SADC region. Harmonisation of registration requirements and joint reviews have reduced the workload for both the pharmaceutical industry and the regulatory authorities. Some of the challenges identified were the lack of a centralised submission and tracking system, and the lack of information for applicants on the process for submission of ZaZiBoNa dossiers/applications in the individual countries, including contact details of the focal person. The establishment of a regional unit hosted in one of the member countries to centrally receive and track ZaZiBoNa dossiers/applications was identified as the best strategy for moving forward in the interim with the long-term goal being the establishment of a regional medicines authority.

**Conclusion:**

There was consensus between the pharmaceutical industry and the regulatory authorities as to the way forward to improve the effectiveness and efficiency of the ZaZiBoNa initiative. Implementation of the recommendations identified in this study will lead to enhanced regulatory performance.

## Introduction

**FIGURE 1:**
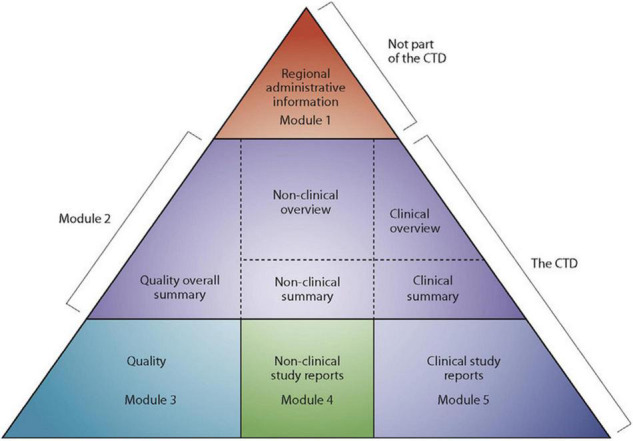
The Common technical document triangle (4).

Medicines and other medical products undergo a rigorous review to ensure compliance with quality, safety, efficacy and local requirements before they are registered in most countries (1, 2). Other factors such as compliance of the manufacturing site(s) with current good manufacturing practices (cGMP) and compliance of product samples with specifications are considered before a medical product is registered by national medicines regulatory authority (1). Traditionally, requirements for registration differed from country to country, which meant that applicants had to compile a new data set each time they wanted to submit their dossiers/applications for registration (2). This presented many challenges in an industry often characterised by multinational operations. The International Conference on Harmonisation of Technical Requirements for Registration of Pharmaceuticals for Human Use (ICH) common technical document (CTD) format, which was finalised in the early 2,000s, addressed this challenge by harmonising the technical requirements for new drug applications (2). The CTD format is made up of 5 modules. Module 1 is region specific; for example, application forms and labels; and it has been acknowledged from the onset that module 1 requirements will be different from country to country. Modules 2–5 are the same across all regions. Module 2 is for overviews and summaries of modules 3–5, module 3 for quality, module 4 for non-clinical study reports and module 5 for clinical study reports (Figure 1) (2–4). Development of the CTD format is a powerful example of the benefits that can come out of collaboration between regulators and the pharmaceutical industry.

### Regulatory Harmonisation in Africa

The CTD format is now used by other countries that are not ICH members (5). The World Health Organization (WHO) prequalification “Guidelines for submission of documentation for a multisource (generic) finished product and preparation of product dossiers in common technical document format” (6) have been adapted or adopted for use by many low- and middle-income countries in the last decade. The CTD format has facilitated harmonisation of medicines registration requirements, work sharing and joint reviews on the African continent (7, 8) as is the case in other emerging markets (5, 9). Established in 2009, the African Medicines Regulatory Harmonisation Initiative (AMRH) is the driving force behind harmonisation of medicines regulation in Africa (10). The AMRH works through the five regional economic blocks recognised by the African Union, for example, Southern African Development Community (SADC), East African Community (EAC) and the Economic Community of West African States (ECOWAS) (11).

### ZaZiBoNa Collaborative Medicine Registration Initiative

ZaZiBoNa is a collaborative medicines registration initiative in the SADC region established in 2013 and formally endorsed by the SADC Health Ministers in 2014 (7). All 16 SADC countries, Angola, Botswana, Comoros Islands, Democratic Republic of Congo, Eswatini, Lesotho, Madagascar, Malawi, Mauritius, Mozambique, Namibia, Seychelles, South Africa, United Republic of Tanzania, Zambia, and Zimbabwe (12), participate in the initiative as either active or non-active members (7). As at December 2021, 333 dossiers/applications had been assessed under the ZaZiBoNa initiative with a median time to recommendation of 12 months (13), which is much shorter than the timelines reported by some of the participating countries for their national procedures (14). Although some feedback on the performance of the initiative has been sought from manufactures through stakeholder meetings in the past, there has not been a comprehensive and structured evaluation of the work-sharing programme for its future direction. Therefore, a study was carried out with the nine active members (regulatory authorities) of the ZaZiBoNa work-sharing initiative to determine their views on its operational effectiveness and efficiency (7). The aim of this study was to evaluate the effectiveness and efficiency of the current operating model of the ZaZiBoNa initiative including the challenges it faces as well as identifying opportunities for improvement from the perspective of applicants.

## Study Objectives

### The Study Objectives Were to

1. Obtain the views of the applicants of the ZaZiBoNa initiative about the performance of the programme to date
2. Identify the challenges experienced by individual applicants since the inception of the ZaZiBoNa initiative
3. Determine the strengths and weaknesses of the initiative
4. Identify the ways for improving the performance of the work-sharing programme
5. Envisage the strategy for moving forward

## Materials and Methods

### Study Participants

Twenty-three applicants who had submitted registration/marketing authorisation applications for both generic and innovator products to the ZaZiBoNa initiative during the period 2017–2021 were identified and invited to participate in the study. Nineteen out of the 23 applicants responded with completed questionnaires, translating to a response rate of 83%. Applicants who submitted applications for registration of generic medicines manufactured outside of the SADC region will be referred to as *Generics (Foreign)* in this report. Applicants who submitted applications for registration of generic medicines manufactured within the SADC region will be referred to as *Generics (Local)*. Applicants who submitted applications for registration of innovator medicines will be referred to as *Innovator*. There were no locally manufactured innovator medicines submitted to ZaZiBoNa in the period under review (2017–2021).

### Data Collection

Data were collected in September 2021 using the Process, Effectiveness and Efficiency rating questionnaire for industry (PEER-IND) developed by the authors. The questionnaire was completed by a representative responsible for ZaZiBoNa submissions in each company. The questionnaire comprised five sections under the headings; Demographics, Benefits of the ZaZiBoNa initiative, Challenges of the ZaZiBoNa initiative, Improving the performance (effectiveness and efficiency) of the work-sharing programme and Envisaging the strategy for moving forward.

To examine the applicability and practicality of the PEER-IND questionnaire, it was piloted with five applicants in August 2021 prior to undertaking the main study. Subsequently, an additional questionnaire was completed by all participants to establish the content validity and relevance of the PEER-IND questionnaire,

### Ethics Committee Approval

The study was approved by the Health, Science, Engineering and Technology ECDA, University of Hertfordshire, United Kingdom [Reference Protocol number: LMS/PGR/UH/04350].

## Results

For the purpose of clarity, the results are presented in five parts, matching the questionnaire sections: Part I—Demographics; Part II—Benefits of the ZaZiBoNa initiative; Part III—Challenges of the ZaZiBoNa initiative; Part IV—Improving the performance of the work-sharing programme; and Part V—Envisaging the strategy for moving forward.

### Part I—Demographics

The study respondents’ age ranged from 33 to 59 years, with a range of regulatory experience from 5 to 30 years. Eleven of the respondents were female and 8 were male. Study participants were classified according to product portfolio and location of manufacturing site. Fifteen (79%) were foreign generic pharmaceutical companies, one (5%) was a local manufacturer of generics and three (16%) were innovator pharmaceutical companies. Of the 333 dossiers/applications assessed as at 31 December 2021, 94% were generics submitted by foreign companies, 5% were new active substances submitted by innovator companies and 1% were generics submitted by the local company.

### Part II—Benefits of the ZaZiBoNa Initiative

#### Benefits of the ZaZiBoNA Initiative

**FIGURE 2:**
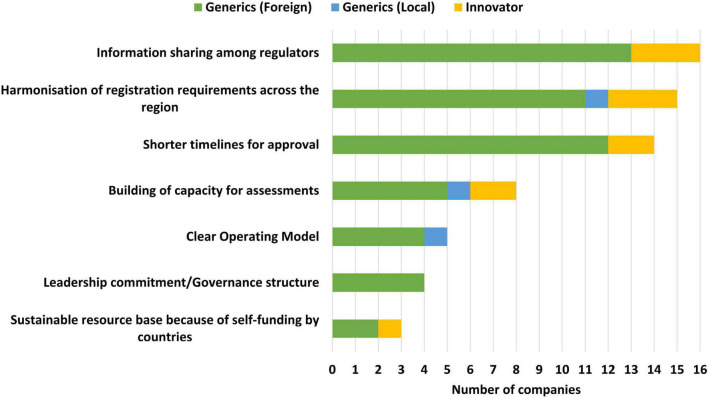
Benefits of the ZaZiBoNa initiative according to pharmaceutical industry respondents.

Information sharing among regulators (16/19), harmonisation of registration requirements across the region (15/19) and shorter timelines for approval (14/19) were identified as the top three benefits of the ZaZiBoNa initiative by the majority of the applicants. However, of note is that less than one third of the applicants believed that the operating model was clear (5/19) or that self-funding by countries created a sustainable resource base for the initiative (3/19) (Figure 2).

#### Benefits of the ZaZiBoNa Initiative to Applicants

**FIGURE 3:**
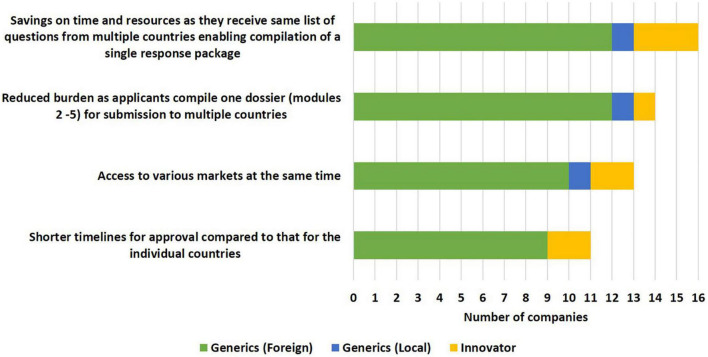
Benefits of the ZaZiBoNa initiative to applicants according to pharmaceutical industry respondents.

The majority of applicants (16/19) viewed the savings of time and resources as a benefit of the initiative, as they received the same list of questions from multiple countries, enabling compilation of a single response package (Figure 3). In addition to this, a large number of applicants (14/19) believed that the burden of compiling several dossiers for different countries was reduced as under ZaZiBoNa they only compiled one dossier (modules 2–5) for submission to multiple countries. Access to various markets at the same time (13/19) and shorter timelines for approval compared with that of the individual countries (11/19) were also identified as benefits to applicants, although some applicants were of the view that ZaZiBoNa timelines of approximately 12 months were comparable to the national timelines for some countries who had improved their timelines in the last 2–3 years.

#### Benefits of the ZaZiBoNa Initiative to Patients

Increased availability of medicines (15/19) and quicker access to quality-assured medicines (14/19) were identified as the benefits of the ZaZiBoNa initiative to patients by the majority of applicants. This was attributed by some applicants to improved commercial viability in otherwise under-resourced territories, resulting from the acceptance/supply of a harmonised medicinal product across the region. However, only 2 out of the 19 applicants believed that the initiative resulted in reduced prices for medicines.

### Part III—Challenges of the ZaZiBoNa Initiative

#### Overall Challenges of the ZaZiBoNa Initiative

The major challenges of the ZaZiBoNa initiative were identified as the lack of centralised submission and tracking (18/19), differences in regulatory performance of the countries (13/19), lack of ability to mandate central registration (12/19) and dependence on the countries’ processes for communication with applicants (12/19). Additional challenges highlighted were the failure by some countries to adhere to the 90 working days set for registration after the ZaZiBoNa recommendation, difficulty following up on dossiers/applications in some countries as there was no clear ZaZiBoNa contact person and the lack of an overall central person in ZaZiBoNa to submit complaints when individual countries were uncooperative.

#### Challenges for Applicants Submitting Applications to the ZaZiBoNa Initiative

**FIGURE 4:**
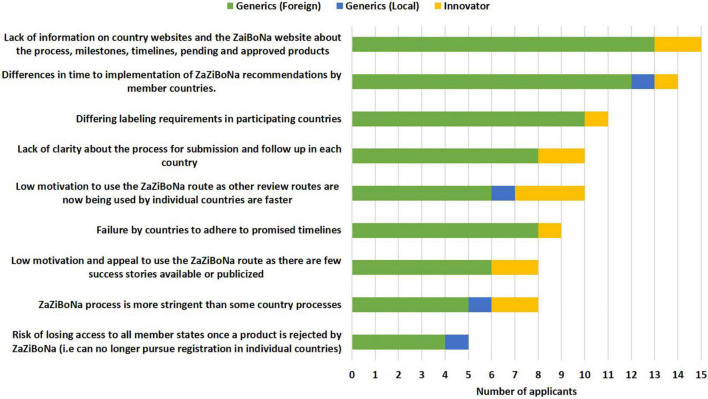
Challenges to the ZaZiBoNa initiative for applicants according to pharmaceutical industry respondents.

The top two challenges faced by applicants in the view of the respondents were lack of information on the country and ZaZiBoNa websites about the process, milestones, timelines and pending and approved medicinal products (15/19) and the differences in time to implementation of ZaZiBoNa recommendations by member countries (14/19). Additional challenges identified by a majority of the applicants were differing labelling requirements in participating countries (11/19), lack of clarity about the process for submission and follow-up in each country (10/19) and low motivation to use the ZaZiBoNa route as other review routes now used by individual countries such as reliance on stringent regulatory authority (SRA) approvals or approvals by other SADC countries were faster (10/19) (Figure 4). The lack of alignment resulting in some of the ZaZiBoNa member countries being more stringent than others was perceived to put smaller companies at a disadvantage compared with larger established companies. Applicants also expressed frustration at having to duplicate efforts in completing WHO forms, which are currently used for ZaZiBoNa and national forms; for example, WHO vs. national Quality Information Summary and Quality Overall Summary.

#### Industry’s Views of the Challenges Faced by Regulators

Industry identified some challenges faced by regulators:

• submission of dossiers and query responses at different times in the member countries, making it difficult to initiate harmonised assessment;
• different internal processes in each of the authorities leading to dissimilar times for adoption of recommendations and processing of query letters and registration certificates;
• inadequate infrastructure and information technology (IT) system and resources;
• unavailability of reliance-related documentation from Stringent Regulatory Authorities (SRA’s) for WHO facilitated SRA reviews;
• difficulty in sharing additional information provided by applicants during submission of responses to respective authorities;
• facilitating various views during the review of a single application by all participating countries;
• limited capacity for the review of bio-therapeutics by some authorities;
• limited number of assessors with adequate skills) available for the ZaZiBoNa process; and
• lengthy assessments and queries due to the combined process and lack of a dedicated team to review the ZaZiBoNa applications.

### Part IV—Improving Performance (Effectiveness and Efficiency)

#### Improving the Effectiveness of the ZaZiBoNa Initiative

**FIGURE 5:**
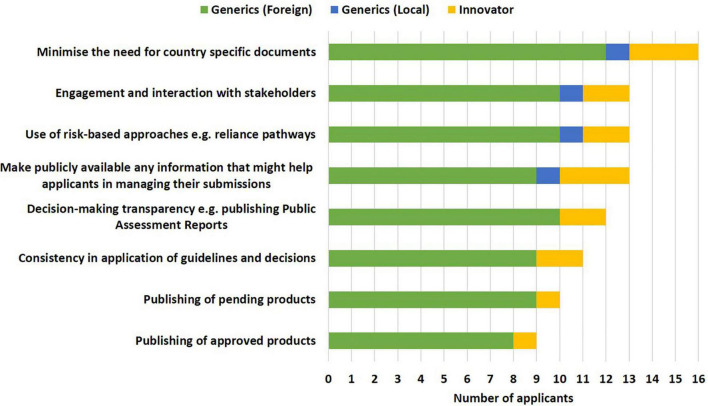
Improving the effectiveness of the ZaZiBoNa initiative according to pharmaceutical industry respondents.

The following approaches, namely minimising the need for country-specific documents (16/19), making publicly available any information that might help applicants in managing their submissions such as document templates, lists of Q&As, timelines and milestones, disclosure of internal standard operating procedures (13/19), use of risk-based approaches such as reliance pathways and engagement (13/19) and interaction with stakeholders (13/19) were selected as the top ways to improve effectiveness of the initiative by the industry. Applicants proposed that having clear communication as to whether a dossier/application has been accepted into the ZaZiBoNa process, the availability of contact details of the focal person in each respective country to enable follow-up of pending dossiers/applications and centralising submission were additional measures that would improve the effectiveness of the initiative (Figure 5).

#### Improving the Efficiency of the ZaZiBoNa Initiative

**FIGURE 6:**
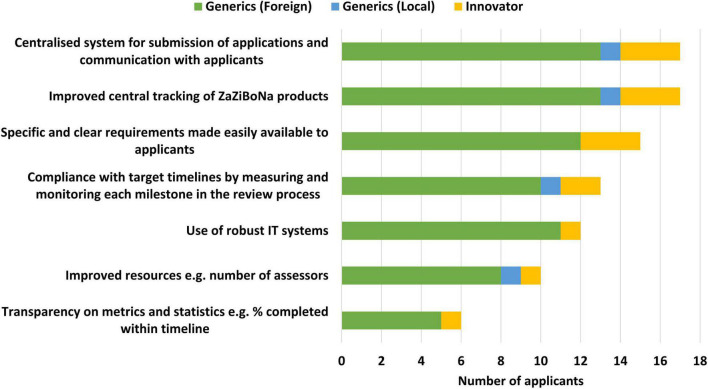
Improving the efficiency of the ZaZiBoNa initiative according to pharmaceutical industry respondents.

Applicants selected improved central tracking of ZaZiBoNa dossiers/applications (17/19) and a centralised system for the submission of applications and communication with applicants (17/9) as the top ways to improve the efficiency of the initiative for applicants. Also identified as contributing to improved efficiency were specific and clear requirements made easily available to applicants (15/19) and compliance with target timelines by measuring and monitoring each milestone in the review process (13/19) (Figure 6).

### Part V—Strategies for Moving Forward

The majority of applicants (15/19) were of the view that the establishment of a regional unit hosted in one of the member countries to centrally receive and track ZaZiBoNa applications was the best strategy moving forward in the interim. The unit would be responsible for allocating work, apportioning the applicable fees to countries, tracking of applications and communication with applicants. The majority of applicants (12/19) were also of the view that to continue with the current operating model was the least effective strategy.

Fifteen out of 19 applicants were of the view that if it were legally possible, the establishment of a SADC regional medicines authority would be the best strategy to address the challenges and areas requiring improvement in the initiative. However, it was acknowledged by some of the applicants that immense legal and administrative hurdles exist in the SADC setting; for example, lack of harmonisation in the regional dossier sections, as well as differences in country-specific registration requirements, which will need to be addressed if a regional authority is to be established. An example of this is the requirement of the South African Health Products Regulatory Authority (SAHPRA) that comparative dissolution studies should be conducted between an SRA oral formulation vs. the local test medicinal product to demonstrate equivalence in three different dissolution media is unique to SAHPRA and different to all other ZaZiBoNa members. A few of the applicants (3/19) were not in support of the establishment of a SADC regional medicines authority, as some of these felt that it would increase the operating costs of the entire evaluation process, which would affect them in the end.

## Discussion

The results of this study show that applicants perceive that there has been a high degree of success and benefit from the ZaZiBoNa initiative for applicants, patients and regulators. A similar study was conducted with regulators (15) and the responses compared. Regulators and industry commonly agreed that information sharing among regulators and harmonisation of registration requirements across the region were the top benefits of the ZaZiBoNa initiative. There was agreement too that as a result, the initiative has saved industry the time and resources spent compiling submissions and responses to queries. Both regulators and pharmaceutical industry were of the view that the initiative has resulted in greater access to quality-assured medicines by patients, although there was a difference in opinion regarding the time that this is taking. A number of applicants were of the view that ZaZiBoNa resulted in shorter timelines, while only a minority of regulators believed that this was a benefit (15). Further investigation is required to understand why the initiative is not resulting in reduced prices of medicines for patients, since both regulators and industry acknowledge that time, resources and the effort required to get medicines approved has been reduced.

While the successes and benefits of the ZaZiBoNa initiative have been examined in this study, it is apparent that there is now a need to review the operating model in order to address the challenges that have been identified to make it more effective and efficient. Views of the regulators (15) and industry were compared and there was agreement on the challenges such as lack of information for applicants on country websites, failure by applicants to meet deadlines for submission of responses, inadequate resources, an unclear operating model and differing performance by participating regulatory authorities.

Interestingly, only a minority of the regulators and industry were of the view that self-funding by countries created a sustainable resource base for this initiative; therefore, there is still a need for partner support or other sources of funding at present. This is supported by studies in the literature highlighting the inadequacy of resources currently available to authorities in low- to middle-income countries (16–20). Challenges highlighted by the industry but not identified in the regulators study (15) are the difficulties faced by applicants when they need to follow up on pending dossiers/applications or seek arbitration in situations in which individual authorities were uncooperative. The challenges identified in this study are not unique to this initiative, as they have been identified for other regions such as the East African Community, with applicants indicating that the goal of harmonisation, which was to ensure quicker access to quality-assured medicines was not always being met (21). Addressing the challenges identified in this study presents a unique opportunity for ZaZiBoNa to re-engineer its operating model, thus ensuring that the initiative remains competitive when compared with the other routes available for registration of medicines.

The removal of country-specific requirements was identified in both this and the regulators study (15) as one of the best ways to improve effectiveness and efficiency. Authorities in the SADC region now require submission of the dossier in CTD format; however, there are some country-specific requirements identified in this study such as bioequivalence, labelling and local Quality Information Summary and Quality Overall Summary that still impede harmonisation efforts and this is consistent with findings from other studies in the literature (15, 22). There is now a need for countries to make a deliberate effort to collectively review their legislation in order to include provisions that facilitate the harmonisation of the registration and labelling requirements for medicinal products in the SADC region.

Although the ZaZiBoNa initiative has been in operation for8 years, the process for submission in some countries remains unclear to applicants (15). This, in addition to a number of other challenges identfied in this study such as failure by some countries to register medicines and issue GMP certificates within the set timelines after a ZaZiBoNa recommendation, can be attributed to the participating authorities having differing capacities (14, 18). Centralised submission and tracking were therefore proposed by both regulators and industry as ways to improve the effectiveness and efficiency of this initiative. This can be achieved through the development of a regional unit hosted in one of the member countries to coordinate submissions. A proposal made by industry, but not identified by regulators, was the need to implement a system that would allow applicants to submit an “expression of interest” to have their dossiers/applications assessed under ZaZiBoNa. This would enable the regulators to adequately plan and allocate resources as well as ensure that applicants are informed from the outset as to whether their dossiers/applications have been accepted for review under ZaZiBoNa. At present, some applicants only become aware that their dossier/application will be reviewed under ZaZiBoNa months after submission. Although some of the participating countries have information on the ZaZiBoNa process on their websites and the contact details of the focal person are known, this is not the case in all the countries and this detracts from the initiative’s effectiveness and efficiency.

### Way Forward

In the long term, the establishment of a regional medicines authority was proposed as a strategy for moving forward. This is not unique to SADC and has also been proposed for other harmonisation initiatives (21, 23, 24). To do this, a binding memorandum of understanding should be developed mandating the establishment of the regional medicines authority. A similar model has been implemented in the Standardisation, Quality Assurance, Accreditation and Metrology (SQAM) Programme in the Southern African Development Community (25). This would ideally make it possible for a SADC-approved medicinal product to be marketed in all the SADC countries. Issues such as the need to strengthen pharmacovigilance systems and to have agreement on the use of labelling that is in the three official SADC languages, English, Portuguese and French, should be considered before implementation as these are important for patient safety. In addition, the issue concern of increased costs to applicants that was raised by a few of the applicants who were not in support of this proposal should also be taken into consideration.

Key recommendations to improve effectiveness and efficiency of ZaZiBoNa work-sharing initiative include:

• **Information for applicants**—Full information on the ZaZiBoNa process including contact details of the focal person, timelines and milestones as well as approved medicinal products should be published on the website of every participating authority and ZaZiBoNA.
• **Submission procedures**—The initiative should introduce expression of interest forms, which will be completed by applicants prior to submission of dossiers. Communication of acceptance for assessment under the ZaZiBoNa initiative or otherwise should be made within a defined period from the date of submission.
• **Information management systems**—The initiative should use automated systems to enable the online tracking of submissions through all the stages of review including information on the meetings at which dossiers/applications are discussed. Applicants should also be able to track their dossiers/applications using the same system.
• **Product life-cycle management**—The initiative should establish a process for review of post approval changes. Variation requirements should be harmonised so that one application can cater for all markets.
• **Reliance**—The WHO-facilitated SRA procedure for ZaZiBoNa has yielded significant results for some applicants and should be promoted and used for more medicinal products.
• **Centralised submission, tracking and communication system**—As an interim measure, a regional unit hosted in one of the member countries should be piloted to centrally receive, track and coordinate ZaZiBoNa dossier submissions. This will address the various challenges faced by the industry with the current operating model such as differences in the time to implementation of the ZaZiBoNa recommendations for assessments and GMP inspections and the lack of a specified person/office to escalate matters in cases in which applicants have challenges with participating countries.
• **Regional medicines authority**—In the long term, a binding memorandum of understanding should be developed mandating the establishment of a regional medicines authority. This would be similar to the model employed for the SQAM programme in the Southern African Development Community. This would ideally make it possible for a SADC-approved medicinal product to be marketed in all the SADC countries. In the meantime, countries should make a deliberate effort to collectively review their legislation, guidelines and processes in order to truly harmonise the registration and labelling requirements for medicinal products in the SADC region.

### Study Limitations

The scope of this study was limited to the ZaZiBoNa initiative’s process and operating model. In future, it would be helpful to get quantitative data to support these views including the actual metrics of the time taken to register the medicinal products in the individual countries after a ZaZiBoNa recommendation. The status of commercialisation and pricing of the medicinal products in the individual countries as well as the factors influencing this could be the subject of a future study.

## Conclusion

This study has enabled an improved understanding of the performance of the ZaZiBoNa initiative from the applicants’ perspective. Applicants have highlighted the benefits of this initiative as well as some of the challenges. Addressing these challenges will lead to enhanced regulatory performance.

## Data Availability Statement

The raw data supporting the conclusions of this article will be made available by the authors, on request.

## Author Contributions

TS designed the study, collected, analysed the data, and wrote the first draft of the manuscript. GM interpreted the results and reviewed subsequent drafts of the manuscript. SW and SS designed the study, interpreted the results, and reviewed subsequent drafts of the manuscript. All authors contributed to the article and approved the submitted version.

## Conflict of Interest

The authors declare that the research was conducted in the absence of any commercial or financial relationships that could be construed as a potential conflict of interest.

## Publisher’s Note

All claims expressed in this article are solely those of the authors and do not necessarily represent those of their affiliated organizations, or those of the publisher, the editors and the reviewers. Any product that may be evaluated in this article, or claim that may be made by its manufacturer, is not guaranteed or endorsed by the publisher.

## Abbreviations

AMRH: African Medicines Regulatory Harmonisation Initiative
CTD: common technical document
EAC: East African Community
ECOWAS: Economic Community of West African States
PEER-IND: Process, Effectiveness and Efficiency Rating questionnaire for industry
SADC: South African Development Community
SQAM: STANDARDISATION, Quality Assurance, Accreditation and Metrology
SRA: stringent regulatory authority
WHO: World Health Organization.
